# Dietary Malondialdehyde Impair Intestinal Health and Fillet Quality of Hybrid Grouper (*Epinephelus fuscoguttatus*♀ *× E. lanceolatus*♂)

**DOI:** 10.3390/ani14223208

**Published:** 2024-11-08

**Authors:** Xuehan Wang, Jiongting Fan, Xiaohui Dong, Shuang Zhang, Qihui Yang, Shuyan Chi, Haitao Zhang, Junming Deng, Beiping Tan

**Affiliations:** 1 College of Fisheries, Guangdong Ocean University, Zhanjiang 524088, China; xuehan98@163.com (X.W.); 2112101099@stu.gdou.edu.cn (J.F.); dongxiaohui2003@163.com (X.D.); zhangshuang198610@126.com (S.Z.); qihuiyang03@163.com (Q.Y.); chishuyan77@163.com (S.C.); 2 Aquatic Animals Precision Nutrition and High Efficiency Feed Engineering Research Centre of Guangdong Province, Zhanjiang 524088, China; 13828263599@139.com; 3 Guangdong Evergreen Feed Industry Co., Ltd., Zhanjiang 524022, China

**Keywords:** malondialdehyde, immune response, fillet quality, intestinal health, hybrid grouper

## Abstract

Aquafeed kept at elevated temperatures and humidity can result in malondialdehyde (MDA) formation, adversely affecting aquafeed quality and triggering negative reactions in fish. To investigate the detrimental effects of dietary MDA on fish, six experimental diets with varying MDA levels (ranging from 0.03 to 17.72 mg/kg, on dietary crude lipid basis) were administered to three replicates of hybrid grouper for 8 weeks. Dietary inclusion of 4.43 mg/kg MDA significantly decreased serum complement 4 content and lysozyme activity, along with intestinal complement 3, complement 4, and immunoglobulin M contents. Furthermore, dietary inclusion of 8.86 mg/kg MDA significantly increased the activities of interleukin-1 receptor-associated kinase, ubiquitin-protein ligase, p38 mitogen-activated protein kinase, and tumor necrosis factor-α, downregulated the relative expression of *Occludin* but upregulated the relative expression of *HSP70* in the hindgut. Additionally, the highest inclusion of MDA (17.72 mg/kg) significantly upregulated the relative levels of pro-inflammatory cytokines (*IL-1β* and *TNF-α*), caused intestinal inflammation, and damaged the intestinal microbial structure and fish fillet texture. This study demonstrated a dose-dependent response of MDA on hybrid grouper. A low dietary dose of MDA (<2.21 mg/kg) exhibited minimal impact on immune response and fillet quality. However, higher inclusion levels (≥4.43 mg/kg) impaired the intestinal health and fillet quality. Consequently, the safety limit for MDA content in the diet for hybrid grouper has been established at 4.43 mg/kg based on dietary crude lipid basis.

## Simple Summary

Aquafeed stored in hot, humid conditions can produce MDA, harming fish health. In a quest to uncover the negative impacts of dietary MDA on fish, we subjected hybrid grouper to a regimen of six experimental diets, each with a different MDA concentration, over an eight-week experimental period. It was found that diets with 4.43 mg/kg MDA lowered immune response, while 8.86 mg/kg increased inflammation. The highest MDA level (17.72 mg/kg) caused intestinal inflammation and damaged fish fillet texture. The study suggests a safety limit for MDA in grouper diets at 4.43 mg/kg, below which immune and fillet quality are minimally affected. However, higher levels harm gut health and fillet quality.

## 1. Introduction

The hybrid grouper (*Epinephelus fuscoguttatus*♀ × *E. lanceolatu*♂) is an important mariculture species in southern China, valued for its high nutritional content, rapid growth rate, and significant economic potential [1]. As a warm-water carnivorous fish, hybrid grouper requires diets that are rich in protein (44–52%) and lipid (9–17%) [2,3] and are predominantly cultivated in tropical and subtropical waters [4]. Consequently, commercial feed for hybrid grouper often encounters elevated temperatures and humidity during transportation and lay-up periods. This exposure can result in fat oxidation in diet, producing a range of potentially harmful oxidation products, including aldehyde, ketone, alcohol, ester, acid, and other compounds [5,6]. These lipid peroxides pose a risk to cellular components such as DNA, unsaturated fatty acids, and protein, inducing oxidative stress and ultimately compromising fish health.

Malondialdehyde (MDA) is commonly used as a biomarker for lipid peroxidation, primarily due to its correlation with rancidity and the peroxidation of fatty acids [7]. MDA contains two chemically active aldehyde groups, which facilitate the oxidative rancidity of fatty acids in phospholipids of animal membranes. Furthermore, MDA can damage cellular proteins by interacting with amino acid residues within peptide chains and can also impair DNA or RNA by crosslinking with their bases. Lipid peroxidation occurs when reactive nitrogen species and reactive oxygen species interact with the polyunsaturated fatty acid residues of phospholipids, resulting in damage to the cell membrane, and ultimately cell death [8]. Consequently, MDA not only serves as an indicator of the degree of oil oxidation in feed but also acts as a harmful component within that feed.

Former research has underscored the adverse impacts of dietary oxidized lipids on fish [9,10]. However, there has been limited emphasis on MDA as a significant product of lipid peroxidation [11]. Our previous studies have shown that the diet of hybrid grouper may produce higher MDA during production, transportation, and storage. A high concentration damaged the gastrointestinal structure and negatively impacted the intestinal digestive and antioxidant functions [12]. Despite this finding, there was still a lack of comprehensive information regarding dietary MDA in hybrid grouper. Additionally, most aquatic researchers primarily focus on assessing the quality dimension to evaluate the impact of MDA on the growth of aquatic animals. However, a comprehensive multi-index and multi-dimensional analysis of MDA’s effects on the health and quality of aquatic animals still requires further investigation. However, there is no study to evaluate the effects of dietary MDA on intestinal immunity and fillet quality of hybrid grouper, which presents challenges for their healthy breeding. Therefore, this study evaluates the impact of varying levels of dietary MDA on the intestinal health and fillet quality of hybrid grouper.

## 2. Materials and Methods

### 2.1. Local and Ethics Statement

In compliance with the guidelines established in the Guidance of the Care and Use of Laboratory Animals in China (GB/T 35892-2018) [13], all animal research was conducted in strict accordance with ethical standards. The Animal Research and Ethics Committee of Guangdong Ocean University (GDOUIACUC-2021-A0207) granted approval for the research protocols implemented in Zhanjiang, China.

### 2.2. Experimental Feed

The formula of the basal diet and its nutritional components are listed in Table 1. Among them, fish meal, soybean meal, and soybean protein concentrate constitute the main source of protein, while soybean oil and soybean lecithin are the main sources of lipids. The preparation of the basal diet involved crushing and sieving the feed ingredients using a 60-mesh screen, followed by a thorough mixing process. Subsequently, soy oil and soy lecithin were added, and the large oil particles were rubbed open and mixed evenly. Then, an appropriate amount of water was added and fully mixed to ensure uniformity. The resulting mixture was extruded through a 2.5-mm mold utilizing a double screw extruder (F-26 type; South China University of Technology, Guangzhou, China). Finally, the pellets underwent natural drying and stored at −20 °C for future use.

Six experimental diets were formulated and named as M0, M1, M2, M4, M8, and M16, respectively. The experimental diets were treated with varying levels of MDA prior to feeding. In accordance with the protocol outlined in GB/T 5009.191-2016 [14], a standard MDA solution was prepared by dissolving 0.315 g of 1,1,1,3-tetraoxypropane (purity ≥ 97%, Shanghai Maclin Biochemical Co., Ltd., Shanghai, China) in distilled water, subsequently diluting it to a final volume of 1000 mL, and storing it at 4 °C. The MDA content was quantified by first extracting the lipids from the diets, followed by measuring the MDA concentration within the lipid fraction. The gradient for MDA content was established based on prior experiments conducted with grass carp [15] and hybrid grouper [6]. The measured MDA content in the M0, M1, M2, M4, M8, and M16 diets were 0.03, 1.11, 2.21, 4.43, 8.86, and 17.72 mg/kg (on dietary crude lipid basis), respectively.

### 2.3. Experimental Animal and Feeding Management

Juvenile hybrid groupers were obtained from a commercial farm located on Donghai Island in Zhanjiang, China. Prior to the commencement of the study, the fish adapted to the conditions by eating the commercial feed (50% protein level, 10% lipid level) for two weeks. All experimental fish underwent a 24-h fasting period. Following this, a total of 540 healthy juvenile fish, each weighing approximately 14.77 ± 0.01 g, were randomly distributed into 18 fiberglass tanks (300 L). Each tank housed 30 fish, with three replicates for every group. During the 8-week feeding period, fish were fed twice (8:00 and 17:00) daily until they appeared satiated. To maintain optimal water quality, tank waste was removed daily using a siphon. The experimental setup utilized a one-way non-circulating flow through water system, with a flow rate of 0.3 m^3^/h. Water temperature was consistently maintained between 26 °C and 30 °C, dissolved oxygen levels were kept above 6 mg/L, pH levels ranged from 7.5 to 8.0, and salinity was maintained between 26‰ and 28‰.

### 2.4. Sample Collection

After the feeding trial, the experimental fish were not given any food for 24 h. Subsequently, the fish were anesthetized using a eugenol solution (1:12,000; Shanghai Reagent Corporation, Shanghai, China). Furthermore, five fish were randomly selected from each tank and blood was taken from the caudal vein with a 1 mL syringe. The blood samples were then centrifuged at 4 °C (805× *g*, 10 min), and the supernatants were stored at −80 °C for serum index analysis. Subsequently, six fish per tank were dissected, and their hindguts were removed. The hindguts were preserved in RNA later (Ambion, Austin, TX, USA) at 4 °C for one day before being transferred to a −80 °C freezer for analysis of relative mRNA expression. The hindgut contents were quickly placed in liquid nitrogen. Subsequently, the samples were stored at −80 °C for intestinal microbial analysis. Finally, muscle samples were obtained from both sides of the dorsal fin post-slaughter, and a prompt assessment of fillet quality was conducted.

### 2.5. Analyses

#### 2.5.1. Immune and Antioxidant-Related Enzymes Activities Analyses

Approximately 1 g of the hindgut sample was homogenized with normal saline in a 1:9 (*w*/*v*) ratio and subsequently centrifuged at 4 °C to obtain the supernatant. Various parameters were assessed, including immunoglobulin M (IgM), alkaline phosphatase (AKP), complement 3 (C3), complement 4 (C4), and lysozyme (LZM) in both serum and hindgut samples. Additionally, levels of nuclear factor-kappa B (NF-κB), phosphatidylinositide 3′-kinase (PI3K), inhibitor of kappa B kinase (IKK), protein kinase B (PKB), interleukin-1 receptor-associated kinase (IRAK), extracellular regulated protein kinases (ERK), ubiquitin-protein ligase (UBPL), c-Jun N-terminal kinase (JNK), interleukin-10 (IL-10), p38 mitogen-activated protein kinase (p38 MAPK), and tumor necrosis factor-α (TNF-α) in the hindgut were measured following the instructions of the detection kit (Nanjing Jiancheng Bioengineering Institute, Nanjing, China)

#### 2.5.2. Real-Time Quantitative PCR Analysis

Hindgut RNA was extracted utilizing the TransZol Up Plus RNA Kit (Beijing TransGen Biotech Co., Ltd., Beijing, China). The integrity of the RNA was confirmed, and the total RNA concentration and purity were assessed through spectrophotometry (A260:280 nm) following 1% agarose gel electrophoresis. Subsequently, cDNA was synthesized from the RNA utilizing the Prime-Script™ RT Reagent Kit (Takara Bio Inc., Tokyo, Japan) according to the provided protocol. Gene-specific primers were designed using Primer5 software (Table 2) based on the GenBank sequence, with *β-actin* selected as the internal reference gene. Real-time PCR was conducted on a high-throughput PCR system (Light Cycler 480, Roche Diagnostics, Switzerland) with a reaction volume of 10 μL. The reaction mixture consisted of 5 μL of 2X SYBR^®^ Green Pro Taq HS Premix II (Accurate Biotechnology (Hunan) Co., Ltd., Changsha, Hunan, China), 3.8 μL of RNase-free water, 1 μL of cDNA, and 0.1 μL each of the upstream and downstream primers for the target gene. The thermal cycling protocol for real-time quantitative PCR included an initial denaturation step at 95 °C for 30 s, followed by 40 cycles of denaturation at 95 °C for 5 s, annealing at 60 °C for 30 s, and a final extension at 50 °C for 30 s. The relative expression of the target gene was quantified utilizing the 2^−ΔΔCT^ method [16].

#### 2.5.3. Analysis of 16S rDNA Sequencing of Intestinal Microflora

DNA was extracted from intestinal content utilizing the MN NucleoSpin 96 Soil DNA kit to determine its mass and concentration. The V3–V4 regions of 16S rDNA were targeted for PCR amplification, employing primer sequences 338F (5′-ACTCCTACGGGAGGCAGCA-3′) and 806R (5′-GGACTACHVGGGTWTCTAAT-3′) [18]. The resulting PCR products were analyzed through agarose gel electrophoresis, and the band of interest was subsequently recovered using the Monarch DNA Gel Recovery Kit. Following this, high-throughput sequencing was performed on the Illumina HiSeq 2500 platform utilizing PE 250 reads (Illumina, San Diego, CA, USA).

#### 2.5.4. Fillet Quality Measurement

Dorsal muscle samples were meticulously extracted, and shaped into cubes measuring 2 × 2 × 1 cm^3^, and subsequently analyzed for various texture indicators, including hardness, elasticity, cohesion, adhesion, and chewiness using the TA.XT plusC texture analyzer (Stable Micro Systems, London, UK). The samples underwent double-cycle compression testing with a flat-bottomed cylindrical probe P/10 at an ambient temperature of 18–20 °C. Additionally, the color of the dorsal muscle was evaluated utilizing a colorimeter (Chroma Meter CR-400, Konica Minolta, Inc., Tokyo, Japan).

### 2.6. Statistical Analysis

All data were presented as mean ± standard error of mean (SEM). Homoscedasticity and normality were confirmed using the Levene and Kolmogorov–Smirnov tests, respectively. Data analysis was performed using SPSS 25.0 for Windows (SPSS Inc., Chicago, IL, USA), employing one-way ANOVA analysis followed by Duncan’s test to compare means between treatments at a remarkable level of *p* < 0.05.

## 3. Results

### 3.1. Immune Response

Dietary MDA content did not affect the serum IgM level and intestinal LZM activity (*p* > 0.05; Table 3). The serum C3 and C4 contents, as well as LZM and AKP activities, gradually depressed with the increase of dietary MDA levels. However, no marked variations were noted in the serum C3 content and AKP activity across the M0-M8 groups or in the serum C4 content and LZM activity across the M0-M2 groups (*p* > 0.05). Similarly, the intestinal C3, C4, and IgM contents, as well as AKP activity, linearly decreased with the increase in dietary MDA content. Significant differences were only found in the intestinal C3, C4, and IgM contents across the M0 and M4-M16 groups and in the intestinal AKP activity across the M0 and M2-M16 groups (*p* < 0.05).

### 3.2. Intestinal Inflammation-Related Factors

Dietary MDA content had no marked impact on the intestinal PKB activity (*p* > 0.05; Table 4). However, the intestinal NF-kB, IKK, PI3K, IRAK, UBPL, ERK, p38 MAPK, JNK, and TNF-α contents linearly increased with the increase of dietary MDA content. Among them, the intestinal NF-kB, IRAK, UBPL, p38 MAPK, and TNF-α contents were greater in the M8 and M16 groups in comparison to the M0 group; the intestinal IKK and ERK contents were greater in the M2-M16 groups in comparison to the M0 group; and the intestinal PI3K and JNK contents were greater in the M16 group in comparison to the M0 group (*p* < 0.05). In contrast, the intestinal IL-10 content showed a decreasing trend with the increase of dietary MDA content, which was lower in the M16 group in comparison to the M0 and M1 groups (*p* < 0.05).

**Figure 1:**
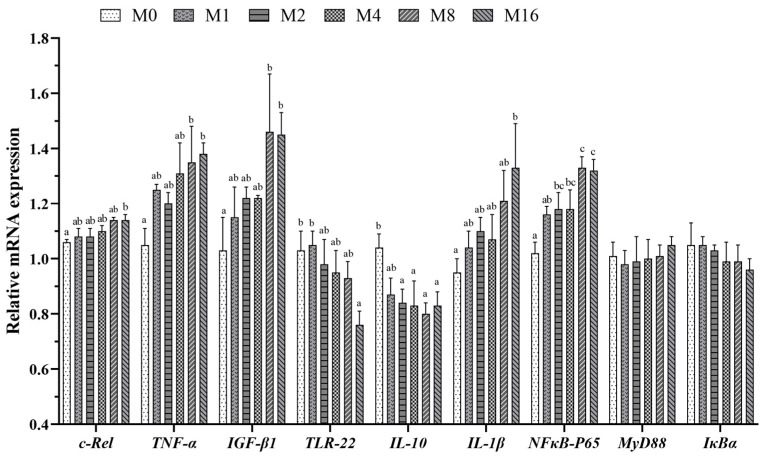
Effect of dietary malondialdehyde level on the relative expression of intestinal inflammation-related factors in hybrid grouper. M0, M1, M2, M4, M8, and M16 represented the dietary groups with 0, 1, 2, 4, 8, and 16 mg/kg malondialdehyde (on dietary crude lipid basis), respectively. Values are means with standard error of three replications represented by vertical bars. a, b, c means with different letters were significantly different (*p* < 0.05).

Similar results were observed for the relative expression levels of the above-mentioned parameters (Figure 1). Dietary MDA content had no significant effect on the relative expression levels of intestinal *MyD88* and *IκBα* (*p* > 0.05). The relative expression levels of *c-Rel*, *TNF-α*, *IGF-β1*, *IL-1β*, and *NF-κB p65* generally up-regulated with the increase of dietary MDA level. Among them, the relative expression levels of *c-Rel* and *IL-1β* were higher in the M16 group compared to the M0 group; the relative expression levels of *TNF-α* and *IGF-β1* were higher in the M8 and M16 groups compared to the M0 group; and the relative expression level of *NF-κB p65* was greater in the M2-M16 groups in comparison to the M0 group (*p* < 0.05). On the contrary, the relative expression levels of *TLR-22* and *IL-10* showed a decreasing trend with the increase of dietary MDA content; the relative expression level of *TLR-22* was lower in the M16 group compared to the M0 group, the relative expression level of *IL-10* was lower in the M2-M16 groups compared to the M0 group (*p* < 0.05).

### 3.3. Intestinal Cell Apoptosis and Tight Junction

**Figure 2:**
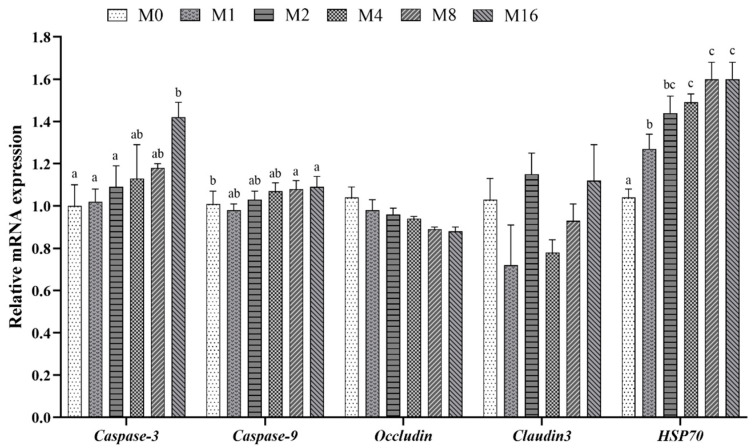
Effect of dietary malondialdehyde level on the relative expression of intestinal apoptosis and tight junction in hybrid grouper. M0, M1, M2, M4, M8, and M16 represented the dietary groups with 0, 1, 2, 4, 8, and 16 mg/kg malondialdehyde (on dietary crude lipid basis), respectively. Values are means with standard error of three replications represented by vertical bars. ^a,b,c^ means with different letters were significantly different (*p* < 0.05).

Dietary MDA content had no marked impact on the relative expression of intestinal *Claudin 3* and *Caspase-9* (*p* > 0.05; Figure 2). With the increase of dietary MDA content, the relative expression level of *Occludin* gradually decreased, while the relative expression levels of *Caspase-3* and *HSP70* gradually increased. Among them, the relative expression level of *Occludin* was markedly lower in the M8 and M16 groups in comparison to the M0 group; the relative expression level of *Caspase-3* was greater in the M16 group in comparison to the M0 group; the relative expression level of *HSP70* was greater in the M4, M8, and M16 groups in comparison to the M0 and M1 groups (*p* < 0.05).

### 3.4. Intestinal Microflora Analysis

Through alpha diversity analysis, the Goods_coverage index for each group surpassed 0.99, suggesting that the sequencing depth adequately represented all species present in the samples. Dietary MDA content had no marked impact on the Shannon index and Simpson index of intestinal microflora (*p* > 0.05; Table 5). However, the Chao 1 and Ace indices were markedly greater in the M4 and M16 groups in comparison to the M0 group (*p* < 0.05).

**Figure 3:**
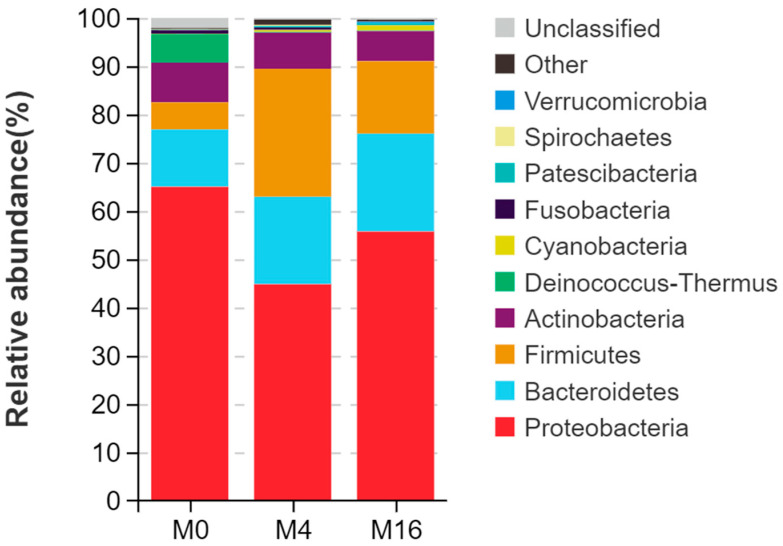
Effect of dietary malondialdehyde level on the intestinal microflora at the phylum level in hybrid grouper. M0, M4, and M16 represented the dietary groups with 0, 4, and 16 mg/kg malondialdehyde (on dietary crude lipid basis), respectively.

The four predominant phyla in the M0 group were Proteobacteria (65.00%), Bacteroidetes (11.89%), Actinobacteria (8.24%), and Firmicutes (5.62%) (Figure 3). The four predominant phyla in the M4 group were Proteobacteria (44.81%), Firmicutes (26.52%), Bacteroidetes (18.13%), and Actinobacteria (7.50%). The four predominant phyla in the M16 group were Proteobacteria (55.73%), Bacteroidetes (20.30%), Firmicutes (15.01%), and Actinobacteria (6.22%). With the increase in dietary MDA content, the abundance of Proteobacteria first decreased and then increased, while the abundance of Firmicutes first increased and then decreased. Additionally, with the increase of dietary MDA content, the abundance of Bacteroidetes generally increased, while the abundance of Actinobacteria generally decreased.

**Figure 4:**
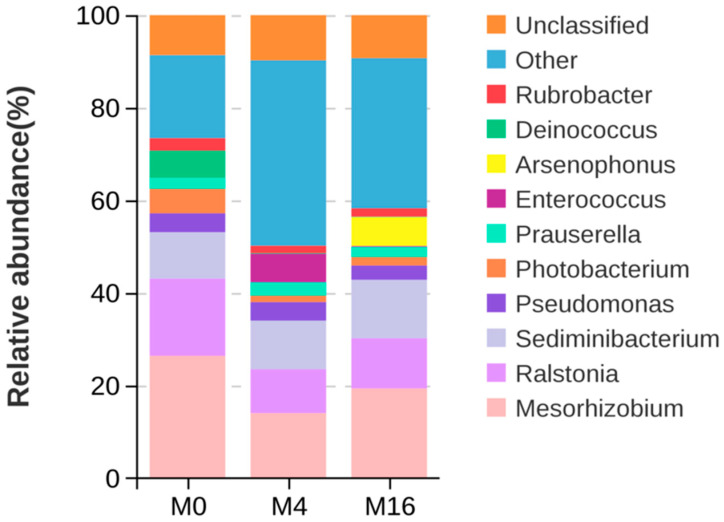
Effect of dietary malondialdehyde level on the intestinal microflora at the genus level in hybrid grouper. M0, M4, and M16 represented the dietary groups with 0, 4, and 16 mg/kg malondialdehyde (on dietary crude lipid basis), respectively.

The six predominant genera in the M0 group were *Mesorhizobium* (26.41%), *Ralstonia* (16.68%), *Sediminibacterium* (10.03%), *Photobacterium* (5.28%), *Pseudomonas* (4.08%), and *Prauserella* (2.37%) (Figure 4). The six predominant genera in the M4 group were *Mesorhizobium* (14.04%), *Sediminibacterium* (10.51%), *Ralstonia* (9.49%), *Pseudomonas* (4.00%), *Prauserella* (2.90%), and *Photobacterium* (1.38%). The six predominant genera in the M16 group were *Mesorhizobium* (19.37%), *Sediminibacterium* (12.61%), *Ralstonia* (10.86%), *Pseudomonas* (3.09%), *Prauserella* (2.19%), and *Photobacterium* (1.83%). With the increase in dietary MDA content, the abundance of *Mesorhizobium*, *Ralstonia*, and *Photobacterium* first decreased and then increased, while the abundance of *Prauserella* first increased and then decreased. Additionally, with the increase of dietary MDA content, the abundance of *Sediminibacterium* generally increased, while the abundance of *Pseudomonas* generally decreased.

### 3.5. Fillet Quality

Dietary MDA content had no marked impact on the adhesiveness and resilience of dorsal muscle (*p* > 0.05; Table 6). However, the hardness, springiness, cohesiveness, gumminess, and chewiness generally increased with the increase of dietary MDA content, which was greater in the M16 group in comparison to the M0 group (*p* < 0.05). Additionally, the muscle L*, a*, and b* values were not affected by dietary MDA content (*p* > 0.05; Table 7).

## 4. Discussion

Storage conditions characterized by elevated temperatures and humidity often lead to increased levels of MDA in aquafeed. This compound not only serves as an indicator of oil oxidation in feed but also presents a significant risk to aquatic organisms [6]. The presence of MDA can adversely affect the feed quality and detrimentally influence the growth performance and overall health of fish. In fish, humoral components such as complements and immunoglobulins are integral to both innate immune and adaptive immune responses. The innate immune system functions as the primary defense mechanism in fish [19], and dietary composition has been shown to impact this innate immune response [20,21]. IgM is the principal mediator of the adaptive immune response, facilitating the production of antibodies that bind specifically to antigens [22]. Additionally, C3 and C4 play critical roles in the destruction of pathogenic microorganisms and are essential components of the innate immune system [23,24]. These proteins are pivotal in regulating inflammation within the fish immune system [25]. Furthermore, AKP contributes to immune response, while LZM is crucial for natural defense mechanisms [26]. LZM is particularly important within the innate immune system due to its antibacterial properties, which involve the cleavage of bacteria by disrupting their cell walls [27]. The present study observed that both C3 and C4 levels, as well as AKP activity, decreased in serum and intestine with increasing dietary MDA content. Similarly, Zhuo et al. [28] demonstrated that the consumption of oxidized fish oil led to a reduction in the AKP activity, thereby compromising the immunity of yellow catfish (*Pelteobagrus fulvidraco*). These findings indicated that elevated MDA levels can induce oxidative stress in fish, highlighting the close relationship between the body’s antioxidant system and its immune function. The impact of dietary MDA content on immunity was mediated through the regulation of these immune factors.

The formation of MDA can disrupt the redox balance within the intestines, leading to an excess of free radicals. This oxidative stress triggers intestinal cells to release inflammatory mediators such as TNF-α and interleukins (IL-1β and IL-6). Consequently, these mediators initiate further inflammatory responses, resulting in cellular infiltration and tissue damage [29]. Importantly, intestinal inflammation is intricately connected to fish immunity [30]. In fish, pro-inflammatory and anti-inflammatory factors generally maintain a dynamic equilibrium; however, an inflammatory response is elicited when this balance is disrupted. IL-10 displays immunosuppressive characteristics by hindering the growth of regulatory T cells, reducing the production of pro-inflammatory molecules, and limiting antigen presentation. At the same time, it facilitates the activation and maturation of mast cells, NK cells, and B cells, while also boosting the phagocytic capability of monocytes and macrophages [31,32]. TNF-α is a multifunctional proinflammatory cytokine involved in mediating inflammation and immune reactions [33]. Notably, it has been reported that serum levels of TNF-α, along with *NF-κB* and *TNF-α* mRNA levels in the liver of Amur minnow (*Rhynchocypris lagowski*), were significantly elevated with increased oxidation of fish oil in the diet, while mRNA levels of *IL-10* in the liver were markedly reduced [34]. The present study demonstrated that an increase in dietary MDA content significantly elevated the mRNA levels of pro-inflammatory cytokines, including *TNF-α*, and *IL-1β*, while concurrently reducing the mRNA levels of anti-inflammatory cytokines such as *IGF-β1* and *IL-10*. These findings suggest that a high dietary MDA intake promotes the onset of intestinal inflammation in hybrid grouper. As a principal regulatory element, NF-κB orchestrates the production of pro-inflammatory factors and enhances the immune response in fish by interacting with key components of the IKK, PI3K, IRAK, UBPL, and ERK signaling pathways [35,36,37]. In this study, MDA was found to upregulate the mRNA levels of *c-Rel* and *NF-κB-P65* within the NF-κB pathway. Furthermore, it was observed that the activities of NF-κB, IKK, PI3K, IRAK, UBPL, ERK, p38 MAPK, JNK, and TNF-α increased, while the activity of IL-10 decreased with rising dietary MDA levels, corroborating the results obtained from fluorescence quantification. Collectively, these findings indicated that high doses of MDA stimulate the NF-κB and MAPK signaling pathways by modulating relevant factors, thereby leading to intestinal inflammation in hybrid grouper and subsequently influencing the fish’s immune response.

MDA is frequently recognized as a significant biomarker of oxidative stress [38] and is considered one of the primary toxic agents contributing to the oxidative rancidity of oils and fats [39]. Initially, these harmful substances interact with the intestinal mucosal tissue [40], where MDA may exacerbate lipid peroxidation of the cell membrane, leading to cellular structural damage and apoptosis [41]. Concurrently, it has been demonstrated that caspases can help maintain cellular stability by regulating both apoptosis and inflammation [42], while the production of HSP70 plays a protective and reparative role for biological cells. Furthermore, Occludin is essential for preserving barrier function between cells and regulating substance permeability. The expression level of *Occludin* directly influences the integrity and functionality of epithelial cells, particularly in the intestinal and vascular endothelial contexts [43]. Previous experimental findings obtained via intestinal transmission electron microscopy suggest that MDA, along with other oxidative products from fish oil, may compromise the integrity of the tight junction structure in grass carp, with MDA identified as the primary active compound [44]. This study revealed a significant correlation between dietary MDA content and the expression levels of key proteins involved in cellular integrity and stress response. As the MDA concentration in the diet increased, the relative expression of *Occludin* progressively decreased, while the levels of *Caspase-3* and *HSP70* exhibited a corresponding increase. Notably, when the MDA level in the feed surpassed 8.86 mg/kg, the manifestations of cellular structural damage and apoptosis became markedly pronounced. These results indicated that elevated MDA concentrations induce apoptosis in intestinal cells and compromise intestinal barrier function, corroborating previous findings. The reduction in *Occludin* level, associated with higher dietary MDA, suggested a detrimental impact on cellular barrier integrity, exacerbating tissue damage and inflammation. Concurrently, the elevated expression of *Caspase-3* and *HSP70* implies that cells responding to high MDA levels initiate apoptosis to eliminate damaged cells as a mechanism to mitigate oxidative stress and associated cellular injury.

The intestinal microflora is frequently underappreciated as a ‘microbial organ’ within animal hosts [45]. These microorganisms exhibit stability within the animal’s digestive system over extended periods, with their reproductive processes dependent on the favorable environment and nutrient supply provided by the host. The intestinal flora is integral to various complex physiological mechanisms of the host and plays a crucial role in gastrointestinal development, digestive function, metabolic balance, and immune response [46,47,48]. Research has demonstrated that the composition and richness of intestinal flora are closely linked to intestinal health, serving as important indicators of the overall health of animals [49,50]. Prolonged stressors can significantly alter the composition of the gut microbiota, and the change in this microbiota increases the sensitivity to intestinal pathogens [51]. Feed is the primary factor influencing the composition of intestinal flora [52]. Previous studies have indicated that when dietary MDA levels exceed 8.86 mg/kg, fish exhibit a significant decline in intestinal digestive capacity, including reduced activity of stomach enzymes such as trypsin, lipase, amylase, and maltase [12]. This experiment demonstrated that varying levels of dietary MDA significantly impacted the abundance of intestinal flora in hybrid grouper, changing the composition of intestinal microflora. At the phylum level, the predominant bacteria in the hindgut during this experiment were Proteobacteria, Bacteroides, and Firmicutes, which were largely consistent with the dominant intestinal flora observed in other studies on grouper [53,54]. Previous studies have suggested that an increase in the abundance of Proteobacteria is a potential diagnostic signature of dysbiosis and risk of disease [55]. In this research, dietary MDA content of 17.72 mg/kg was associated with an increased abundance of Proteobacteria, which contributed to an imbalance in the intestinal flora and subsequent intestinal inflammation in hybrid grouper. Firmicutes are known to enhance fish digestion and immunity, thereby mitigating the effects of pathogens [19]. Additionally, Actinobacteria play a crucial role in promoting nutrient absorption and inhibiting harmful bacteria within the intestine [56]. However, our findings indicated that at a dietary MDA level of 17.72 mg/kg, there was a notable decrease in the abundance of both Actinobacteria and Firmicutes. This decline facilitated the colonization of harmful bacteria in the intestine, thereby diminishing the host’s resistance. At the genus level, the study revealed that a dietary MDA content of 17.72 mg/kg led to an increase in the abundance of *Mesorhizobium*, *Ralstonia*, and *Photobacterium*. The reduction in beneficial bacteria directly correlated with the increase in these potentially harmful genera within the intestinal microbiota of hybrid grouper. Notably, some species of *Photobacterium* are regarded as opportunistic pathogens in fish; while they typically do not threaten healthy hosts, they can induce infections when the host’s immune system is compromised or damaged [57]. Consequently, the dietary MDA content of 17.72 mg/kg disrupts the intestinal flora structure in hybrid grouper. This situation results in a decrease in beneficial bacteria and an increase in harmful ones, leading to inflammation in the intestines.

Lipid oxidation products generate free radicals, which subsequently lead to protein oxidation and disrupt the protein structure. This disruption ultimately compromises the quality of aquatic products [58]. MDA is the primary secondary product of lipid oxidation, and its concentration has a direct influence on the protein oxidation process [59]. Elevated MDA levels can significantly affect the nutritional quality and sensory characteristics of fish, particularly their texture. The texture of fish muscle is a critical criterion for assessing the quality of aquatic products [60,61]. Hardness relates to the adhesion of fish flesh, which helps maintain its shape; cohesion pertains to the strength of intercellular binding forces; and elasticity and restorative properties reflect the biological elasticity of the fish body. Greater muscle hardness is associated with increased muscle fiber density, which in turn affects meat quality [60]. Furthermore, oxidized lipids can adversely impact the quality of fish by altering the flavor profiles of nucleoproteins, free amino acids, and volatile flavor compounds [62]. In this study, an increase in dietary MDA content was associated with improvements in the hardness, elasticity, cohesion, adhesion, and chewiness of the dorsal muscles in hybrid grouper. These findings indicated that a high dose of MDA in feed can enhance the texture of the dorsal muscle, thereby influencing its taste. Specifically, elevated levels of MDA may result in more compact fish meat, leading to increased hardness due to alterations in muscle fiber structure or water loss [63]. Conversely, the color of the dorsal muscle is primarily influenced by the concentration of myoglobin, fat-soluble pigments (such as carotenoids), and other pigments [64]. Changes in muscle color not only affect market value but are also closely linked to the nutritional quality of fish. In this study, however, dietary MDA content did not significantly impact the color of the dorsal muscles in hybrid grouper. This suggested that the hybrid grouper possesses a certain physiological adaptability that enables it to manage oxidative stress. Even with variations in MDA content, the fish’s physiological mechanisms appear to stabilize the color of the dorsal muscle. Notably, this stability persists even when dietary MDA content reaches 17.72 mg/kg, indicating no effect on the color of the dorsal muscle.

## 5. Conclusions

A low dietary dose of MDA (<2.21 mg/kg) exhibited minimal effects on both the immune response and fillet quality of hybrid grouper. In contrast, an excessive MDA content (≥4.43 mg/kg) correlated with a rise in detrimental intestinal bacteria and a deterioration of intestinal barrier function. This led to cell apoptosis, inflammation, and a compromised immune response in hybrid grouper. Additionally, a high dose of dietary MDA (≥4.43 mg/kg) negatively impacted the texture and taste of fish fillets. Consequently, the safety limit for MDA content in hybrid grouper feed has been established at 4.43 mg/kg.

## Figures and Tables

**Table 1:** Ingredients and chemical composition of the experimental diets (% dry matter).

Ingredients	%
Fish meal	45.00
Soybean protein concentrate	21.00
Soybean meal	6.00
Wheat flour	17.91
Soybean oil	5.30
Soybean lecithin	2.00
Ca(H_2_PO_4_)_2_	1.20
Choline chloride	0.40
Vitamin C	0.03
Compound premix ^1^	1.00
Cellulose microcrystalline	0.16
Proximate composition	
Dry matter (DM, %)	92.34
Crude protein (% DM)	49.62
Crude lipid (% DM)	11.07
Ash (% DM)	16.95

^1^ Compound premix was supplied by Beijing Enhalor Biotechnology Co., Ltd., Beijing, China (g kg^−1^ mixture): vitamin A, 500,000 IU; vitamin D3, 100,000 IU; vitamin E, 4.00 g; vitamin K3, 1.00 g; vitamin B1, 0.50 g, vitamin B2, 1.00 g; vitamin B6, 1.00 g; vitamin B12, 0.002 g; nicotinic acid, 4.00 g; calcium pantothenate, 2.00 g; biotin, 0.01 g; folic acid, 0.10 mg; vitamin C, 15.00 g; ferrum, 10.00 g; cuprum, 0.30 g; zinc, 5.00 g; manganese, 1.20 g; iodine, 0.08 g; cobalt, 0.02 g; selenium, 0.03 g.

**Table 2:** Primer pair sequences used in real-time PCR.

Gene	Primer Sequence	Genbank Accession No.
*Caspase-3*	F-CGCAAAGAGTAGCGACGGA	[17]
	R-CGATGCTGGGGAAATTCAGAC	
*Caspase-9*	F-TTTTCCTGGTTATGTTTCGTGG	[17]
	R-TTGCTTGTAGAGCCCTTTTGC	
*Occludin*	F-GGAGGAGAAACAGGGAATGAACT	KF861990.1
	R-TCTGCTACAGCCTGGTATTTGG	
*Claudin3*	F-AGCCTTCATCGGCAGCAA	EU714179.1
	R-GGATGCCTCGTCGTCAATG	
*HSP70*	F-ATCAATCCAGACGAGGCA	AY423555.2
	R-TACCCAGGGACAGAGGC	
*IκBα*	F-TGTTCTCGCTGACCAATCCTG	KF773745.1
	R-CCACTGAATGTCATCATAACCCAC	
*c-Rel*	F-GTATTCAATCAGCCCTACCAACC	EF363482.1
	R-GGCGTGGCAGATTGTTCATAG	
*MyD88*	F-TCCTCTCGTCGCCCTGAAT	GQ202584.1
	R-CCGCTTTGGTGGGGTTTAC	
*TNF-α*	F-AGACATCACGACGCAATCAGG	KC981238.1
	R-GCCTTGACCGTTTCTCCACTC	
*IGF-β1*	F-CCGCTTCATCACCAACGAG	GQ205390.1
	R-CCGCTCATCCTCATTTCCTT	
*TLR-22*	F-CGAGCCAGGTAAACCCATCA	HQ456304.1
	R-CTCATCAAACAGGCGGAAGC	
*IL-10*	F-AAGCAAACGACGACTTGGACA	KJ741852.1
	R-GCAGCACCGTGTTCAGATAAAA	
*IL-1β*	F-ATGCCTGAGGGACTGGAACTT	EF582837.1
	R-CTCATCAGTCGGTGGAGTTGC	
*NF-κB p65*	F-CAACGACACCACTAAGACCCAC	EU219847.1
	R-GTCACCAATGAGATGCGAACA	
*β-actin*	F-GGCTACTCCTTCACCACCACA	AY510710.2
	R-TCTGGGCAACGGAACCTCT	

*Caspase-3*, cysteinyl aspartate specific proteinase-3; *Caspase-9*, cysteinyl aspartate specific proteinase-9; *HSP70*, heat shock protein 70; *IκBα*, inhibitor kappa B alpha; *MyD88*, myeloid differentiation primary response protein 88; *TNF-α*, tumor necrosis factor-α; *IGF-β1*, insulin-like growth factor-β-1; *TLR-22*, toll-like receptor 22; *IL-10*, interleukin-10; *IL-1β*, interleukin-1-β; *NF-κB p65*, nuclear factor kappa-B-p65; *β-actin*, beta-actin.

**Table 3:** Effect of dietary malondialdehyde level on the immune response of hybrid grouper.

	Diets						*Pr > F* ^†^		
	M0	M1	M2	M4	M8	M16	ANOVA	Linear	Quadratic
Serum									
Complement 3 (g/L)	0.12 ± 0.01 ^b^	0.12 ± 0.00 ^ab^	0.12 ± 0.00 ^ab^	0.11 ± 0.01 ^ab^	0.11 ± 0.00 ^ab^	0.10 ± 0.01 ^a^	0.04	0.05	0.16
Complement 4 (g/L)	0.19 ± 0.01 ^c^	0.18 ± 0.00 ^bc^	0.17 ± 0.01 ^bc^	0.16 ± 0.01 ^ab^	0.15 ± 0.00 ^a^	0.14 ± 0.01 ^a^	<0.01	<0.01	<0.01
Immunoglobulin M (μg/mL)	35.28 ± 1.18	34.12 ± 0.81	34.87 ± 2.80	33.51 ± 0.67	33.67 ± 1.04	31.64 ± 2.52	0.73	0.09	0.24
Lysozyme (U/L)	5.55 ± 0.09 ^c^	5.56 ± 0.61 ^c^	5.17 ± 0.28 ^bc^	4.20 ± 0.04 ^ab^	3.98 ± 0.29 ^ab^	3.76 ± 0.11 ^a^	0.03	<0.01	<0.01
Alkaline phosphatase (U/mL)	3.52 ± 0.43 ^b^	3.39 ± 0.20 ^ab^	3.24 ± 0.22 ^ab^	2.84 ± 0.31 ^ab^	2.80 ± 0.20 ^ab^	2.59 ± 0.06 ^a^	0.01	0.01	0.02
Intestine									
Complement 3 (mg/g protein)	43.37 ± 2.68 ^c^	41.38 ± 2.74 ^c^	38.95 ± 4.97 ^bc^	30.92 ± 1.57 ^ab^	26.00 ± 2.39 ^a^	26.80 ± 0.80 ^a^	<0.01	<0.01	<0.01
Complement 4 (mg/g protein)	80.66 ± 2.42 ^b^	75.22 ± 7.00 ^b^	66.05 ± 6.33 ^ab^	57.44 ± 3.01 ^a^	57.10 ± 6.73 ^a^	53.18 ± 1.68 ^a^	0.01	<0.01	<0.01
Immunoglobulin M (mg/g protein)	15.68 ± 0.47 ^c^	15.50 ± 1.49 ^c^	14.77 ± 0.95 ^bc^	12.55 ± 0.81 ^ab^	11.52 ± 0.55 ^a^	11.63 ± 0.71 ^a^	0.01	<0.01	<0.01
Lysozyme (U/g protein)	2.05 ± 0.03	2.15 ± 0.17	2.00 ± 0.22	1.89 ± 0.24	1.86 ± 0.03	1.58 ± 0.20	0.31	0.01	0.05
Alkaline phosphatase (U/g protein)	1.43 ± 0.08 ^c^	1.14 ± 0.24 ^bc^	0.99 ± 0.16 ^b^	0.94 ± 0.08 ^b^	0.85 ± 0.07 ^b^	0.41 ± 0.05 ^a^	<0.01	<0.01	<0.01

^a,b,c^ Values are means ± standard error of three replications (*n* = 3). Means with different superscripts were significantly different (*p* < 0.05). M0, M1, M2, M4, M8, and M16 represented the dietary groups with 0, 1, 2, 4, 8, and 16 mg/kg malondialdehyde (on dietary crude lipid basis), respectively. ^†^ Significance probability associated with the F-statistic.

**Table 4:** Effect of dietary malondialdehyde level on the intestinal inflammation-related factors in hybrid grouper.

	Diets						*Pr > F* ^†^		
	M0	M1	M2	M4	M8	M16	ANOVA	Linear	Quadratic
NIK (U/g protein)	37.66 ± 1.93 ^a^	44.33 ± 2.64 ^ab^	45.17 ± 3.70 ^ab^	46.45 ± 2.29 ^ab^	48.34 ± 3.73 ^b^	48.14 ± 2.53 ^b^	0.04	0.06	0.05
IKK (U/μg protein)	0.16 ± 0.01 ^a^	0.17 ± 0.00 ^ab^	0.19 ± 0.01 ^bc^	0.21 ± 0.00 ^c^	0.21 ± 0.01 ^c^	0.21 ± 0.00 ^c^	<0.01	<0.01	<0.01
PI3K (U/mg protein)	0.11 ± 0.00 ^a^	0.12 ± 0.00 ^ab^	0.12 ± 0.01 ^ab^	0.12 ± 0.00 ^ab^	0.12 ± 0.00 ^ab^	0.13 ± 0.01 ^b^	0.02	0.02	0.05
PKB (U/g protein)	88.57 ± 9.37	95.36 ± 5.15	81.80 ± 1.01	74.13 ± 4.74	82.20 ± 8.78	75.42 ± 1.21	0.27	0.12	0.23
IRAK (U/kg protein)	86.70 ± 6.11 ^a^	102.12 ± 6.20 ^ab^	111.48 ± 5.51 ^ab^	111.08 ± 10.25 ^ab^	114.47 ± 7.27 ^b^	127.36 ± 10.52 ^b^	<0.01	0.01	0.01
UBPL (U/g protein)	47.97 ± 3.73 ^a^	53.00 ± 1.45 ^ab^	52.38 ± 1.60 ^ab^	54.55 ± 2.11 ^ab^	56.52 ± 3.04 ^b^	56.93 ± 0.88 ^b^	0.02	0.02	0.03
ERK (U/g protein)	18.51 ± 1.50 ^a^	23.42 ± 1.23 ^ab^	25.65 ± 2.51 ^b^	27.59 ± 2.19 ^b^	27.83 ± 3.31 ^b^	28.60 ± 1.03 ^b^	0.04	0.02	<0.01
p38 MAPK (U/g protein)	63.56 ± 6.29 ^a^	68.70 ± 4.69 ^a^	75.33 ± 3.23 ^ab^	85.86 ± 7.86 ^ab^	91.61 ± 9.60 ^b^	95.80 ± 3.87 ^b^	0.04	<0.01	<0.01
JNK (U/g protein)	32.58 ± 4.10 ^a^	33.17 ± 2.89 ^a^	33.70 ± 1.68 ^ab^	38.62 ± 1.97 ^ab^	39.85 ± 1.82 ^ab^	41.74 ± 0.80 ^b^	<0.01	<0.01	<0.01
TNF-α (ng/mg protein)	16.00 ± 0.86 ^a^	17.29 ± 2.30 ^ab^	20.13 ± 2.89 ^ab^	19.22 ± 2.41 ^ab^	22.91 ± 0.66 ^b^	23.81 ± 0.38 ^b^	<0.01	<0.01	0.01
IL-10 (ng/mg protein)	47.79 ± 2.76 ^b^	46.88 ± 2.23 ^b^	41.33 ± 7.13 ^ab^	36.34 ± 3.39 ^ab^	36.10 ± 1.70 ^ab^	31.69 ± 0.22 ^a^	<0.01	<0.01	<0.01

^a,b,c^ Values are means ± standard error of three replications (*n* = 3). Means with different superscripts were significantly different (*p* < 0.05). M0, M1, M2, M4, M8, and M16 represented the dietary groups with 0, 1, 2, 4, 8, and 16 mg/kg malondialdehyde (on dietary crude lipid basis), respectively. NIK, NF-kappa B inducing kinase; IKK, inhibitor of kappa B kinase; PI3K, phosphatidylinositide 3-kinase; PKB, protein kinase B; IRAK, interleukin receptor-associated kinase; UBPL, ubiquitin-protein ligase; ERK, extracellular regulated protein kinases; p38 MAPK, p38 mitogen-activated protein kinase; JNK, c-Jun N-terminal kinase; TNF-α, tumor necrosis factor-alpha; IL-10, interleukin-10. ^†^ Significance probability associated with the F-statistic.

**Table 5:** Effect of dietary malondialdehyde level on the alpha diversity of intestinal microflora in hybrid grouper.

	M0	M4	M16
Goods_coverage	0.99 ± 0.00	0.99 ± 0.00	0.99 ± 0.00
Chao	299.03 ± 57.97 ^a^	636.70 ± 38.54 ^b^	624.62 ± 29.62 ^b^
Ace	304.09 ± 56.31 ^a^	679.59 ± 63.75 ^b^	674.10 ± 23.85 ^b^
Shannon	4.25 ± 0.31	5.75 ± 0.53	5.12 ± 0.69
Simpson	0.87 ± 0.03	0.94 ± 0.00	0.92 ± 0.03

^a,b^ values are means ± standard error of three replications (*n* = 3). Means with different superscripts were significantly different (*p* < 0.05). M0, M4, and M16 represented the dietary groups with 0, 4, and 16 mg/kg malondialdehyde (on dietary crude lipid basis), respectively.

**Table 6:** Effect of dietary malondialdehyde level on the texture of dorsal muscle in hybrid grouper.

	Diets						*Pr > F* ^†^		
	M0	M1	M2	M4	M8	M16	ANOVA	Linear	Quadratic
Hardness	0.95 ± 0.07 ^a^	0.99 ± 0.05 ^ab^	0.96 ± 0.06 ^a^	0.96 ± 0.05 ^a^	1.06 ± 0.06 ^ab^	1.18 ± 0.08 ^b^	<0.01	0.08	0.03
Adhesiveness	−11.29 ± 2.74	−11.54 ± 2.43	−14.33 ± 1.80	−13.77 ± 1.76	−16.91 ± 2.60	−14.24 ± 2.13	0.56	0.15	0.34
Springiness	0.23 ± 0.02 ^a^	0.21 ± 0.02 ^a^	0.26 ± 0.03 ^ab^	0.28 ± 0.03 ^ab^	0.31 ± 0.04 ^ab^	0.34 ± 0.04 ^b^	<0.01	0.03	<0.01
Cohesiveness	0.10 ± 0.00 ^a^	0.10 ± 0.00 ^a^	0.09 ± 0.01 ^a^	0.10 ± 0.01 ^ab^	0.11 ± 0.02 ^ab^	0.13 ± 0.00 ^b^	0.03	0.21	0.17
Gumminess	85.85 ± 13.39 ^a^	99.58 ± 17.67 ^a^	110.41 ± 6.55 ^ab^	104.12 ± 12.83 ^ab^	116.62 ± 8.14 ^ab^	133.17 ± 3.48 ^b^	0.02	<0.01	<0.01
Chewiness	20.75 ± 0.75 ^a^	21.34 ± 2.84 ^a^	26.08 ± 7.08 ^ab^	31.68 ± 8.18 ^ab^	35.87 ± 6.33 ^ab^	44.26 ± 7.36 ^b^	<0.01	0.02	<0.01
Resilience	0.04 ± 0.00	0.04 ± 0.00	0.04 ± 0.00	0.04 ± 0.00	0.04 ± 0.01	0.05 ± 0.00	0.35	0.07	0.07

^a,b^ values are means ± standard error of three replications (*n* = 3). Means with different superscripts were significantly different (*p* < 0.05). M0, M1, M2, M4, M8, and M16 represented the dietary groups with 0, 1, 2, 4, 8, and 16 mg/kg malondialdehyde (on dietary crude lipid basis), respectively. ^†^ Significance probability associated with the F-statistic.

**Table 7:** Effect of dietary malondialdehyde level on the color of dorsal muscle in hybrid grouper.

	Diets						*Pr > F* ^†^		
	M0	M1	M2	M4	M8	M16	ANOVA	Linear	Quadratic
L*	59.52 ± 1.40	58.81 ± 1.55	57.79 ± 1.47	57.90 ± 0.39	56.08 ± 0.63	56.39 ± 0.62	0.30	0.04	0.05
a*	−3.94 ± 0.13	−3.74 ± 0.28	−3.23 ± 0.30	−3.88 ± 0.16	−3.45 ± 0.46	−3.37 ± 0.10	0.30	0.18	0.35
b*	6.43 ± 0.29	7.47 ± 0.60	6.71 ± 0.29	6.70 ± 0.50	8.03 ± 1.03	8.04 ± 0.23	0.27	0.05	0.12

Values are means ± standard error of three replications (*n* = 3). M0, M1, M2, M4, M8, and M16 represented the dietary groups with 0, 1, 2, 4, 8, and 16 mg/kg malondialdehyde (on dietary crude lipid basis), respectively. ^†^ Significance probability associated with the F-statistic.

## Data Availability

The data supporting this study’s findings can be obtained upon request from the corresponding author.
